# Supplementary material for: Dynamic Mechanical Control of Alginate-Fibronectin Hydrogels with Dual Crosslinking: Covalent and Ionic
Source: Polymers (Basel). 2021 Jan 29;13(3):433. doi: 10.3390/polym13030433 (PMC7866402; doi:10.3390/polym13030433)
Supplement: Supplementary file 1 [file polymers-13-00433-s001.pdf]

Supplementary Material

Dynamic mechanical control of alginate-fibronectin hydrogels with dual crosslinking: covalent and ionic

Sara Trujillo [1,2], Melanie Seow [1,4], Aline Lueckgen [4], Manuel Salmeron-Sanchez [1,2,3]* and Amaia Cipitria [5]*

1 Centre for the Cellular Microenvironment, University of Glasgow, Glasgow, UK
2 Centre for Biomaterials and Tissue Engineering (CBIT), Universitat Politècnica de València, Camino de Vera s/n, 46022 Valencia, Spain
3 Biomedical Research Networking Centre in Bioengineering, Biomaterials and Nanomedicine (CIBER-BBN), Valencia, Spain
4 Julius Wolff Institute & Centre for Musculoskeletal Surgery, Charité - Universitätsmedizin Berlin, Berlin 13353, Germany
5 Max Planck Institute of Colloids and Interfaces, Department of Biomaterials, 14476 Potsdam, Germany

Correspondence: Manuel.Salmeron-Sanchez@glasgow.ac.uk (M.S.-S.); amaia.cipitria@mpikg.mpg.de (A.C.)

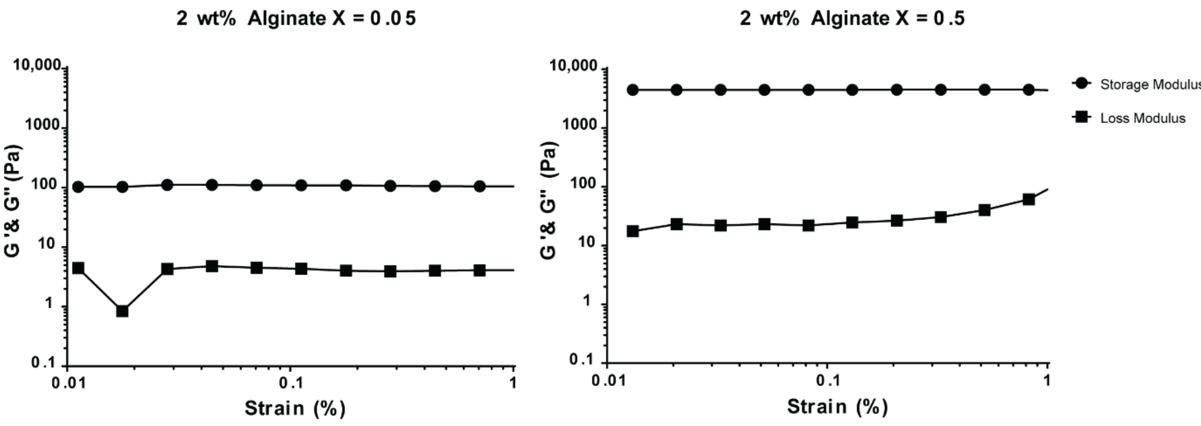

Figure S1. Representative strain sweep curves at an angular frequency of 10 rad·s^{-1} depicting the storage (G′) and loss (G″) modulus of 2wt% alginate hydrogels polymerised with low and high crosslinking ratio (X=0.05 and X=0.5).
